# Advances and Challenges in Forensic Genetics

**DOI:** 10.3390/genes17101215

**Published:** 2026-09-30

**Authors:** Venusia Cortellini, Noemi Argirò

**Affiliations:** Institute of Legal Medicine, University of Brescia, 25123 Brescia, Italy; noemi.argiro@unibs.it

Forensic genetics has changed substantially over the past decades. Conventional short tandem repeat (STR) profiling remains central for human identification (HID), but the field has now gathered a much broader range of analytical approaches, including massively parallel sequencing (MPS), genome-wide single nucleotide polymorphism (SNP) analysis, DNA methylation and epigenetic biomarkers, advanced kinship inference, and increasingly sophisticated statistical frameworks. These developments have increased both the quantity and type of information that can be recovered from biological evidence, including highly compromised samples. However, the forensic value of these increasingly complex data still depends on factors such as sample condition, the specific question addressed, the availability of reference data, and the statistical models used for interpretation. At the same time, increasing analytical power brings additional challenges related to validation, bioinformatic analysis, population representation, and their translation into routine forensic practice. This Special Issue brings together six original articles and two reviews that address these issues from different, but closely connected, perspectives.

Degraded DNA remains one of the main challenges in forensic genetics. Zupanič Pajnič reviews the effects of environmental and postmortem factors on DNA preservation and discusses the use of STRs, SNPs, mitochondrial DNA, and NGS when conventional profiling becomes less effective [1]. Rather than proposing a single replacement for STR analysis, the review emphasizes the need to select markers and analytical strategies according to sample quality and the forensic question. A more specific laboratory problem is addressed by Porcu et al., who evaluated an optimized DNA extraction protocol for putrefied yellow bone marrow [2]. In the sample examined, the modified protocol produced higher DNA concentrations and allele counts than the standard procedure, including complete autosomal STR profiles in several samples that yielded very limited profiles with the standard extraction.

In the framework of skeletal remains, Thomas et al. validated a 95K SNP capture panel together with the Parabon Fx platform for extended kinship analysis in historical US military remains [3]. The method produced useful kinship information, up to third-degree and, in some cases, fourth-degree relationships, even from highly degraded skeletal samples, and extended the range of possible family reference samples. At the same time, the study illustrates some of the interpretative difficulties that emerge with distant relationships.

A broader view of skeletal identification is provided by Zupanič Pajnič and Leskovar [4]. Their review considers genetic, epigenetic, isotopic, proteomic, and histological methods within a preservation-guided framework. Different techniques contribute different types of information, from sex and kinship to age, diet, mobility, and geographic origin, and their usefulness depends on which biological material has survived. Since no single method can be expected to provide all the information required for identification, the review therefore supports a coordinated, preservation-guided use of complementary methods.

Questions of relatedness are also addressed from a different perspective by Connell et al., who examined Y-STR mutation and male differentiation across extended pedigrees [5]. They showed that paternal relatives separated by a number of meioses may differ at more than one Y-STR locus, with a large proportion of changes occurring at rapidly mutating markers. This has practical implications for the identification of historical remains. In such situations, Y-STR mismatches should be interpreted considering the number of meioses and locus-specific mutation rates rather than treated as an automatic exclusion.

Challenges can also occur in the opposite direction, such as when two individuals are genetically almost indistinguishable. The limits of genetic differentiation are particularly clear in a study by Meyer et al. on monozygotic twins [6]. Shotgun sequencing of a blood trace and a buccal reference sample from one twin identified candidate single-nucleotide variants, but these were not confirmed by targeted sequencing. The lack of a reference sample from the second twin and the use of different tissue types were important study limitations. However, the study provides a practical and reproducible workflow combining shotgun sequencing with targeted validation of candidate variants.

Population data remain fundamental to the statistical interpretation of forensic DNA evidence. Djeliova et al. reported allele frequencies and forensic parameters for 16 autosomal STR loci in 220 unrelated individuals from South-West Bulgaria [7]. Their study provides regional reference data for identification and kinship calculations and confirms the high informativeness of several loci, particularly SE33. More generally, it illustrates why appropriate population databases remain necessary for reliable likelihood ratio calculations.

The final contribution addresses interpretation at a different level. Laurent et al. propose a dynamic likelihood ratio framework for DNA database matching based on fast Fourier transform calculations [8]. Instead of relying on fixed locus-count or LR thresholds, the method adjusts the decision threshold according to shared loci, database size, and an accepted false-positive risk. Simulations showed how static criteria become problematic when the number of comparisons grows. The authors describe the framework as a mathematical proof of concept with the need for future validation on operational databases.

The contributions present in this Special Issue cover different parts of the forensic genetics workflow, but several practical requirements emerge. New analytical tools can increase sensitivity and extend the range of questions that can be addressed, yet their usefulness depends on validation, suitable reference data, and statistical models. Future developments should therefore focus not only on generating more genetic information, but also on improving how that information is evaluated, combined with other evidence, and translated into routine forensic practice.

## Data Availability

No new data were created or analyzed in this study. Data sharing is not applicable to this article.
